# Evidence of multiple bacterial, viral, and parasitic infectious disease agents in *Mastomys natalensis* rodents in riverine areas in selected parts of Zambia

**DOI:** 10.1080/20008686.2024.2441537

**Published:** 2024-12-31

**Authors:** Samuel Munalula Munjita, Annie Kalonda, Benjamin Mubemba, Manu Vanaerschot, Cristina Tato, Lusajo Mwakibete, John Tembo, Simbarashe Chitanga, Katendi Changula, Masahiro Kajihara, Walter Muleya, Hirofumi Sawa, Ayato Takada, Matthew Bates, Sody Munsaka, Edgar Simulundu

**Affiliations:** a Department of Biomedical Sciences, School of Health Sciences, University of Zambia, Lusaka, Zambia; b Department of Wildlife Sciences, Copperbelt University, Kitwe, Zambia; c Chan-Zuckerberg Biohub, San Francisco, CA, USA; d HerpeZ, University Teaching Hospital, Lusaka, Zambia; e Department of Preclinical Studies, School of Veterinary Medicine, University of Namibia, Windhoek, Namibia; f Department of Paraclinical Studies, School of Veterinary Medicine, University of Zambia, Lusaka, Zambia; g Division of International Research Promotion, International Institute for Zoonosis Control, Hokkaido University, Sapporo, Japan; h Hokudai Centre for Zoonosis Control in Zambia, University of Zambia, Lusaka, Zambia; i Department of Biomedical Sciences, School of Veterinary Medicine, University of Zambia, Lusaka, Zambia; j One Health Research Center, Hokkaido University, Sapporo, Japan; k International Collaboration Unit, International Institute for Zoonosis Control, Hokkaido University, Sapporo, Japan; l Global Virus Network, Baltimore, MD, USA; m Institute for Vaccine Research and Development (HU-IVReD), Hokkaido University, Sapporo, Japan; n Division of Global Epidemiology, International Institute for Zoonosis Control, Hokkaido University, Sapporo, Japan; o Joseph Banks Laboratories, School of Life and Environmental Sciences, University of Lincoln, Lincolnshire, UK; p Macha Research Trust, Choma, Zambia

**Keywords:** Semen, Rodents, Hotspots, Zoonoses, Riverine areas, Zambia

## Abstract

**Background:**

Infectious disease agents pose significant threats to humans, wildlife, and livestock, with rodents carrying a third of these agents, many linked to human diseases. However, the range of pathogens in rodents and the hotspots for disease remain poorly understood.

**Aim:**

This study evaluated the prevalence of viral, bacterial, and parasitic pathogens in *Mastomys natalensis* rodents in riverine and non-riverine areas in selected districts in Zambia.

**Methods:**

The study applied metagenomic next generation sequencing (mNGS). Tissues analysed included semen, foetal tissues, and blood-rich organs (liver, spleen, kidneys, and lungs). A multivariate logistic regression model explored the relationship between pathogen presence and host or ecological factors.

**Results:**

A total of 182 rodents were captured, and 14 pathogens were detected in 10.4% of the samples (19/182). Detected organisms included zoonoses (*Klebsiella michiganensis*, *Klebsiella pneumoniae*, *Salmonella enterica*, and *Bartonella elizabethae*); Emerging zoonoses (*Elizabethkingia miricola*, *Klebsiella variicola*, *Bartonella tribocorum,* and Cardiovirus B); among others (*Eimeria papillata* etc). Riverine areas showed higher odds of pathogen presence (OR = 8.45; *p* < 0.001; 95% CI: 3.07–23.26).

**Conclusion:**

These results suggest that M. natalensis harbours multiple infectious agents with zoonotic potential, and riverine regions may be key hotspots for rodent-borne pathogens in Zambia.

## Introduction

Infectious disease agents of animal origin are believed to cause over one billion cases of illness in humans every year [1]. Approximately 33% of the infectious diseases agents (viruses, bacteria, fungi, helminths, and protozoa) found in rodents across the globe are capable of causing diseases in humans [2]. In Africa, Lassa virus (LASV), which causes Lassa fever, and *Yersinia pestis*, responsible for plague, are two of the most well-known pathogens directly linked to rodents [3,4]. Potentially, rodents may also harbour a significant share of the estimated 10,000 viruses lying undisturbed in tropical forests mainly in Africa and Asia [5–7]. Rodents effectively transmit pathogens to humans through contaminated faeces, aerosols, and urine [8] as well as through the consumption of rodent meat [4]. Their efficiency in spilling over pathogens is supported by their close contact with humans, as they live commensally and semi-commensally in human dwellings and surrounding vegetation [8].

The genetic and ecological characterization of rodent-borne pathogens is largely inadequate, as evident in the limited profiling across various studies [9–22]. This gap also extends to understanding the impact of zoonotic pathogens on human health, especially in low-income settings. In these contexts, the lack of prioritization for clinical and laboratory diagnosis of zoonotic diseases further limits comprehensive knowledge of their effects [23]. For example, knowledge about the genetic diversity and ecology of clinically relevant extended-spectrum beta-lactamase (ESBL) producing *Klebsiella* sp in rodents is still in its infancy in Africa [23]. Studies focused on detecting multiple organisms in a single sample are rare in Zambia [24] and throughout Africa [18]. Furthermore, the relationship between pathogen prevalence in rodents and host characteristics, such as sex, is also poorly understood [24]. Similarly, while a connection between high pathogen prevalence—particularly mammarenaviruses and hantaviruses—and riverine areas, swampy regions, and areas with high rainfall has been suggested, it is not yet fully understood [24–29]. This limited understanding hampers efforts to identify the full spectrum of rodent-borne pathogens, pinpoint regions with high pathogen burden, and unravel potential transmission routes and spill-over mechanisms. [29]

Amid these challenges, there has been growing interest in identifying hotspots for zoonotic pathogens, coupled with the emergence of metagenomic next-generation sequencing (mNGS), which has revitalized efforts to explore the eco-epidemiology of pathogens of wildlife-origin [24,29–31]. The mNGS technology enables broader and efficient screening for multiple pathogens in a single sample within 24 hours [32–35]. In the present study, we aimed to investigate the presence of pathogens of varying zoonotic potential and associated risk factors in wild *Mastomys natalensis* rodents in riverine and non-riverine areas in selected districts in Zambia using mNGS-based tools.

## Materials and methods

### Study sites, rodent trapping, and processing

**Figure 1.**
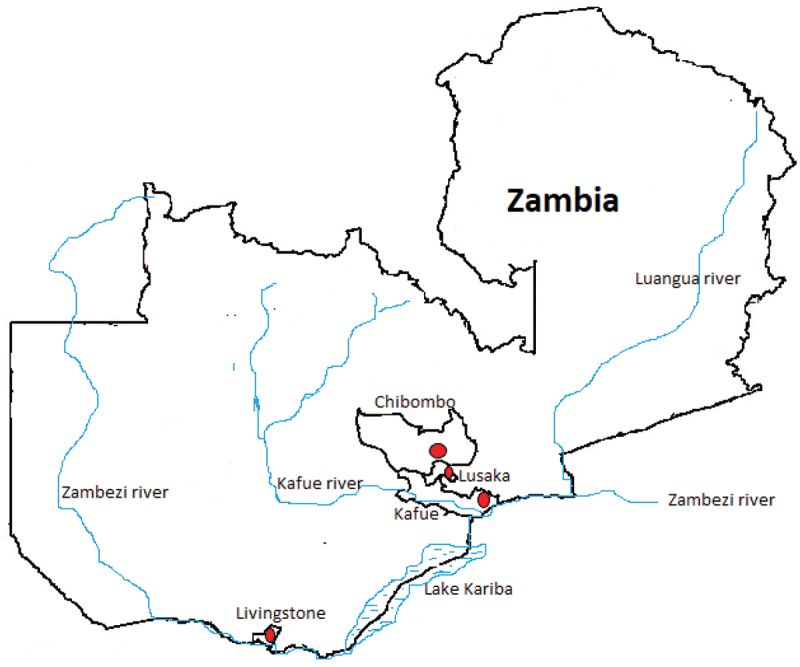
Map of Zambia showing the study sites (red dots) in Chibombo, Kafue, Livingstone and Lusaka.

The study sites were riverine areas in Lusaka and Kafue districts as well as non-riverine areas in Kafue, Livingstone, and Chibombo districts in Zambia (Figure 1). The rodents were captured between July 2019 and March 2022 using Sherman traps (H.B. Sherman, Inc., Tallahassee, FL, USA) and cage traps. In riverine areas, traps were set along the edges of rivers and streams and sometimes within dry river beds. In non-riverine areas, traps were set on the edges of fallow fields. Since the approach was not systematic, some traps were set closer to the rodent holes and along the paths created by the rodents. On each marked rectangular site in riverine or non-riverine areas, 50 to 100 traps were set over 5 nights. Distance between traps was not considered. Traps were baited with peanut butter and pieces of cabbage or carrots and set overnight. Traps were emptied the following morning. Diethyl ether on a dampened piece of cotton wool was used to euthanise the captured animals. The body weight and length of the tail for each animal were measured. Age was determined by relative body size and sexual maturity (perforated vagina in females and external visibility of testicles in males). Animals were classified as juvenile or adult. This was followed by harvesting of the liver, kidneys, seminal vesicles (male accessory sex glands that provide seminal fluid for sperms), spleen, and lungs. Foetuses were also harvested from the gravid uteri of females. The liver, spleen, lung and kidneys from each animal were pooled and packed in one specimen tube for processing. Seminal vesicles and foetuses were packed in separate tubes. The samples were transported in portable cool boxes with ice packs from the study sites to the laboratory. The samples were kept at −30°C until nucleic acid extraction.

### Identification of rodents

Briefly, rodents were identified morphologically by considering fur colour, length of the ear, head-to-body length, tail length, and length of the hind foot. The field guide to mammals of Southern Africa was used as a reference [24,36]. The identity of *Mastomys natalensis*, a rodent known to harbour most mammarenaviruses in Africa, was confirmed by cytochrome *b* mNGS sequences.

### RNA extraction

Approximately 2 grams of each of the liver, kidney, spleen, and lung tissues from a single rodent were cut, pooled, and homogenised in 1 mL phosphate-buffered saline (PBS) in a 1.2 mL tissue specimen tube. To extract semen, a stored seminal vesicle was cut open to release semen into the tissue specimen tube. For pregnant rodents, each foetus was carefully removed from the gravid uterus using forceps before being entirely homogenised. Homogenisation was undertaken in a bench-top tissue homogenizer followed by centrifugation at 1,500 rpm for one minute. Total RNA was extracted from semen and the supernatant of the homogenised tissues using the QIAamp Viral RNA Mini Kit (Qiagen, Hilden, Germany) according to the manufacturer’s instructions. To prevent the degradation of RNA, 60 µL of RNA was mixed with equal volumes of RNA later (ThermoFisher Scientific, Waltham, MA USA) and transported to the Chan-Zuckerberg (CZ) Biohub (CZ-Biohub) in San Francisco, United States of America (USA). At the CZ-Biohub, RNA was re-extracted using the QIAamp Viral RNA Mini Kit (Qiagen, Hilden, Germany) according to the manufacturer’s instructions.

### Metagenomic next generation sequencing

The protocol for library preparation was adapted from the NEBNext Ultra II RNA Library Preparation (non-directional) protocol (New England Biolabs, Ipswich, MA, USA) with slight modifications of the reaction volumes to half the original volumes previously demonstrated and reported in detail elsewhere [24]. We have no reason to think that this modification systematically affected sequences generated in this study. Sequence assembly was conducted using De Novo sequencing technology. Following sequencing, genomic data focused on available sections of the genes of pathogens from the Illumina Novaseq 6000 sequencer was analysed in the CZ ID pipeline [37]. Reads for each sample were subjected to quality control checks which included a set of filters where nucleotide reads per million were to be greater than 100 (NT_rPM >100). The Z score was set at one (NT_z score = 1). The Z-score statistic enabled application of a background model to remove taxa that may have been present in water controls or by-passed filtration [24]. Pathogens were identified from FASTA/FASTQ files within the pipeline.

### BLAST search and phylogenetic analysis of pathogens

FASTA file sequences containing specific genetic information (obtained by chance as the result of broad mNGS sequencing) for each detected pathogen were compared with reference sequences using the Basic Local Alignment Search Tool (BLAST) [38]. Settings were set to default. The highest percent identity score against reference genomes was recorded after the BLAST search. To understand the evolutionary relationships of pathogens reported in this study, phylogenetic analyses were conducted. Reference sequences from GenBank with at least 90% nucleotide sequence identity to the query sequence from this study were included in the phylogenetic tree. The evolutionary relationships were inferred using the maximum likelihood method under a specific probabilistic evolutionary model in Molecular Evolutionary Genetics Analysis Version 11 (MEGA11) [39]. Replicates were restricted to 1000 per tree except where the tree topology required further investigations to come up with a fitting tree. To select the appropriate model, each data set consisting of aligned sequences was subjected to an initial model selection in MEGA11. A model with the lowest BIC (Bayesian Information Criterion) score was considered the best to describe the substitution pattern [39]. Gene specific nucleotide sequences for some of the pathogens generated in this study were deposited in GenBank.

### Statistical analysis

Data analysis was conducted in SPSS Ver. 21 (IBM Corp., Armonk, NY, USA). Prevalence was calculated as the number of rodents positive for a particular pathogen divided by the total number of animals in the study multiplied by 100. A stepwise binary logistic regression model was used to identify the correlates of the presence of any pathogen in rodents as previously demonstrated elsewhere [24]. The Hosmer–Lemeshow test and the Omnibus test were used to test for goodness of fit and predictability, respectively. Independent variables were considered as risk factors when *p-value was* less than 0.05 (*p* < 0·05).

## Results

### Demographic characteristics of captured animals

A total of 182 *Mastomys natalensis* rodents were captured in Lusaka, Kafue, Livingstone, and Chibombo districts in Zambia. The representative mitochondrial cytochrome *b* gene (*cyt b*) sequence confirming the identity for *M. natalensis* was deposited in GenBank under accession number OP778190 as previously reported [24]. Among the 182 *M. natalensis*, 54.9% (100/182) were females compared to 45.1% (82/182) males. A high proportion of *M. natalensis* rodents, 64.3% (117/182), were from riverine areas in Lusaka (101/182) and Kafue (16/182) districts. The remaining 35.7% (65/182) were captured from non-riverine areas in Kafue (5/182), Livingstone (27/182), and Chibombo (33/182) districts. A single *M. natalensis* rodent was pregnant with six fully grown foetuses.

### Landscape of pathogens detected in *M. natalensis*

Fourteen [14] pathogens, besides mammarenaviruses and helminths published earlier [24], were detected in 10.4% (19/182) of *M. natalensis* rodents (Table 1). A notable 94.7% (18/19) of the positive rodents were from riverine areas compared to 5.3% (1/19) from non-riverine areas. Among the positive cases, 31.6% (6/19) of the rodents harboured the pathogens and/or potential pathogens in semen. Spatially, the distribution of infected rodents in a riverine area in Lusaka showed the presence of a cluster on the southern side while in Kafue district, the distribution appeared homogeneous.

**Table 1.** Prevalence of pathogens by tissue type, habitat, and location.

Pathogen	Tissue type	Habitat	Location (District)	Prevalence % (Infected)	GenBank Accession numbers
Cardiovirus B	Pooled	Non-riverine	Chibombo	0.5 (1/182)	PP049374
*Klebsiella michiganensis*	Foetal	Riverine	Lusaka	0.5 (1/182)	OR976489
*Klebsiella pneumoniae*	Semen	Riverine	Lusaka	0.5 (1/182)	OR976488
	Pooled		Lusaka	0.5(1/182)	OR976485
*Klebsiella variicola*	Semen	Riverine	Lusaka	0.5 (1/182)	PP087384
*Salmonella enterica*	Semen	Riverine	Lusaka	1.1 (2/182)	OR976516
*Rickettsia* sp.	Semen	Riverine	Lusaka	0.5(1/182)	PP033731
*Elizabethkingia miricola*	Pooled	Riverine	Lusaka	0.5 (1/182)	OR976518
*Bartonella triboculum*	Pooled	Riverine	Lusaka	0.5(1/182)	–
*Bartonella elizabethae*	Semen	Riverine	Lusaka Lusaka	0.5(1/182)	OR978278
	Pooled			0.5 (1/182)	OR978259
*Helicobacter* sp.	Pooled	Riverine	Lusaka	1.1(2/182)	OR977562 OR977563
*Mycoplasma* sp.	Pooled	Riverine	Lusaka	0.5 (1/182)	PP033726
*Trypanosoma kuseli*	Pooled	Riverine	Lusaka	0.5 (1/182)	PP036896
*Trypanosoma otospermophili*	Pooled	Riverine	Lusaka	0.5(1/182)	PP036897
*Eimeria papillata*	Pooled	Riverine	Lusaka	0.5 (1/182)	OR977712

Pooled = mix of liver, kidney, spleen, and lung tissues per rodent.

### BLAST search and phylogenetic analysis of detected pathogens

#### Viruses

**Figure 2.**
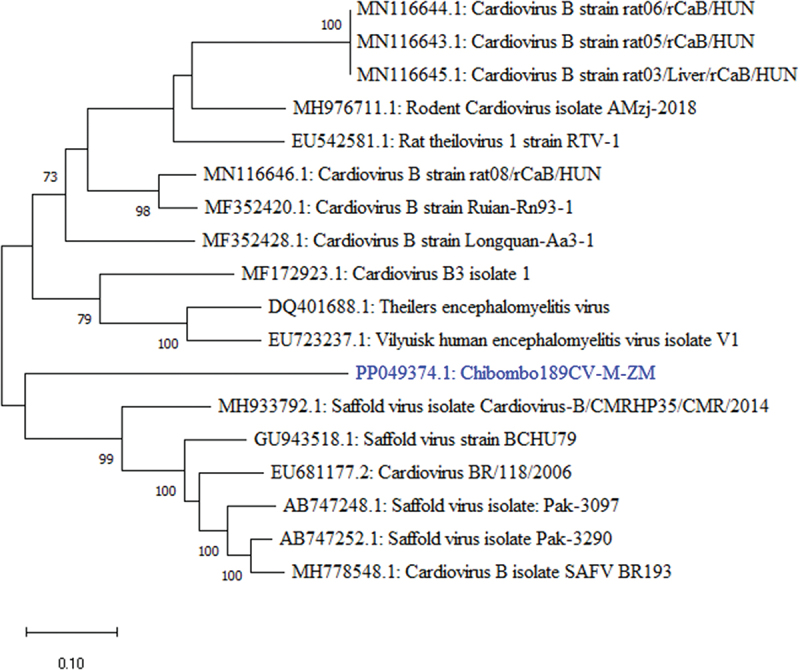
Phylogenetic tree showing evolutionary relationships of cardiovirus from Zambia and reference sequences based on the L segment of the polyprotein gene. The 386 nucleotide long cardiovirus sequence characterised in this study is shown in blue. Numbers at branch nodes represent bootstrap values ≥60% (based on 1000 replicates).

One viral sequence beside previously reported Mammarenaviruses [24] was detected in this study. BLAST search of the leader peptide (L) segment of the polyprotein gene of Chibombo189CV-M-ZM sequence showed 77.29% nucleotide sequence similarity to Cardiovirus B strain rat06/rCaB (GenBank: MN116646.1) from rodents. Further, BLAST search of the translated partial polyprotein amino acid sequence of Chibombo189CV-M-ZM revealed 81.25% amino acid sequence similarity to Cardiovirus B strain Longquan-Aa3–1 (GenBank: AWK02687.1) from rodents. A phylogenetic tree constructed from a partial 386 nucleotide long L gene segment of the larger polyprotein gene sequence revealed that Chibombo189CV-M-ZM formed a distinct lineage sharing the same ancestors with Saffold viruses (Figure 2) which are known to infect humans [40].

#### Bacteria

**Figure 3.**
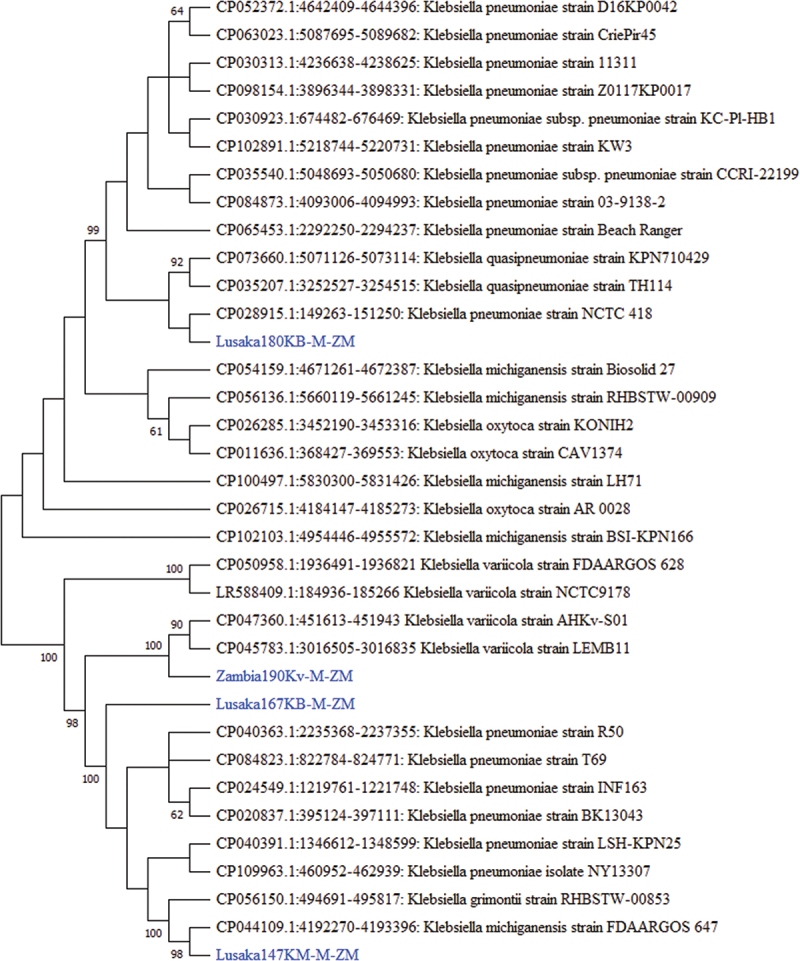
Phylogenetic tree showing evolutionary relationships of the 23S *rRNA* gene segment of *Klebsiella* sp (~1761 nucleotides in length) from Zambia and reference sequences. The sequences characterised in this study are shown in blue text. Numbers at branch nodes represent bootstrap values ≥60% (based on 2000 replicates). Scale bar represents number of nucleotide substitutions per site.

Nine bacterial rRNA sequences, some with established zoonotic potential, were detected in this study (Table 1). A BLAST search of the 23S rRNA gene of Lusaka180KB-M-ZM and Lusaka167KB-M-ZM showed 99.65% and 99.94% nucleotide sequence similarity to *Klebsiella pneumoniae*/NCTC418 (GenBank: CP028915.1) isolated from humans and *Klebsiella pneumoniae*/R50 (GenBank CP040363.1) isolated from rabbits, respectively. Phylogenetic analysis of the 1761 nucleotide long 23S rRNA gene segment of Lusaka180KB-M-ZM showed that it was closely related to *Klebsiella pneumoniae* strain NCTC 418 isolated from a human patient at Johns Hopkins Hospital in the USA (Genbank: CP028915.1) (Figure 2). On the other hand, the Lusaka167KB-M-ZM sequence clustered with a number of strains of *Klebsiella pneumoniae* from rabbits in China (Figure 2). The 23S rRNA nucleotide sequences of Lusaka147KM-M-ZM and Lusaka190Kv-M-ZM were 99.40% and 99.91% similar to *Klebsiella michiganensis*/FDAARGOS/USA (GenBank: CP044109.1) from a child and *Klebsiella variicola*/AHKv-S01/USA (GenBank: CP047360.1) from plant and animal-related sources, respectively. On the phylogenetic tree, the Lusaka147KM-M-ZM sequence formed a separate cluster with *K. michiganensis* strain FDAARGOS-647, a clinical isolate from a sick child in the United States of America (Genbank CP044109.1) (Figure 2). Meanwhile, Lusaka190Kv-M-ZM was closely related to *K. variicola* strain LEMB11 pathogen [41] isolated from food spices in the United Kingdom (Genbank: CP045783.1) and *K. variicola* stain AHKv-S01 from chicken embryos in China (Genbank: CP047360.1) (Figure 3).

**Figure 4.**
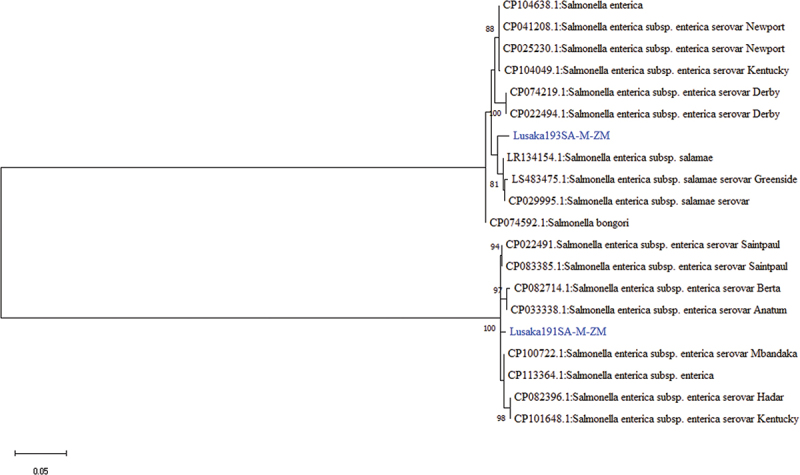
Phylogenetic tree showing evolutionary relationships of the 23S *rRNA* gene segment of *Salmonella* sp (~388 nucleotides in length) from Zambia and reference sequences. The sequences characterised in this study are shown in blue text. Numbers at branch nodes represent bootstrap values ≥60% (based on 2000 replicates). Scale bar represents number of nucleotide substitutions per site.

Among the sequences that were related to salmonella in this study, the 23S rRNA gene sequences of Lusaka193SAL-M-ZM and Lusaka191SAL-M-ZM revealed 98.44% and 99.74% nucleotide sequence similarity to *Salmonella enterica enterica* subsp. salamae of an unknown source (GenBank: LR134154.1) and *Salmonella enterica enterica* subsp. enterica serovar Anatum from pork meat (GenBank: CP118633.1), respectively. Phylogenetically, the 23S rRNA gene sequence of Lusaka193SAL-M-ZM was closely related *to Salmonella enterica enterica* subsp. salamae (GenBank: LR134154.1) (Figure 4), while Lusaka191SAL-M-ZM formed its own lineage in between two clusters that included established zoonoses i.e *Salmonella enterica enterica* subsp. enterica serovar Anatum from pork meat (GenBank: CP118633.1) and *Salmonella enterica* subsp. enterica serovar Mbandaka.

**Figure 5.**
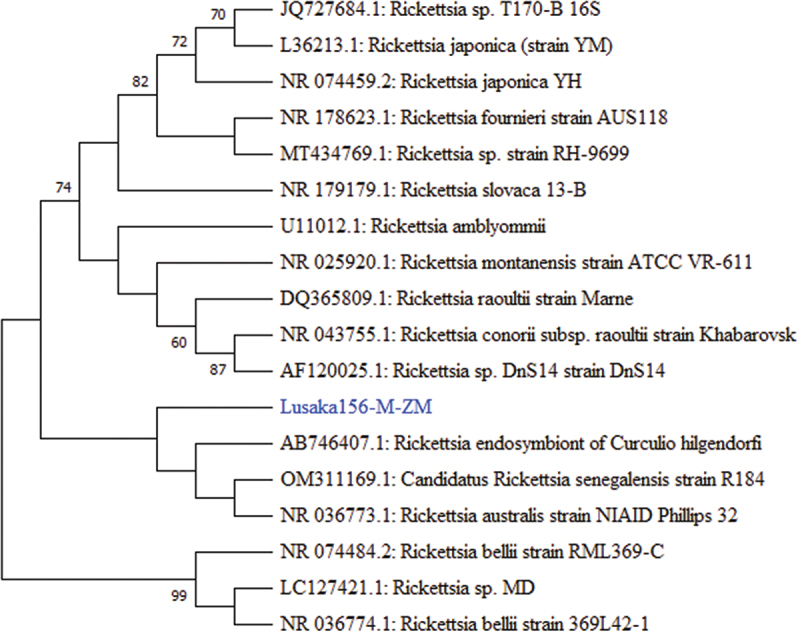
Phylogenetic tree showing evolutionary relationships of the *16S rRNA* gene segment of *Rickettsia* sp (~419 nucleotides in length) from Zambia and reference sequences. The sequence characterised in this study is shown in blue text. Numbers at branch nodes represent bootstrap values ≥60% (based on 1000 replicates). Scale bar represents number of nucleotide substitutions per site.

The only nucleotide sequence related to *Rickettsia* sp. detected herein, Lusaka190RCT-M-ZM *16S rRNA*, was 100% similar to several species of *Rickettsia* by BLAST search, top of which was *Rickettsia japonica* strain LA16/2015, a spotted fever group *Rickettsia* detected in a human in China (GenBank CP047359.1). However, phylogenetic analysis of the said sequence, (Lusaka190RCT-M-ZM), indicated a close relationship to a cluster of *Rickettsia* sp of unknown zoonotic potential and *Rickettsia australis,* a known zoonoses (Figure 5).

**Figure 6.**
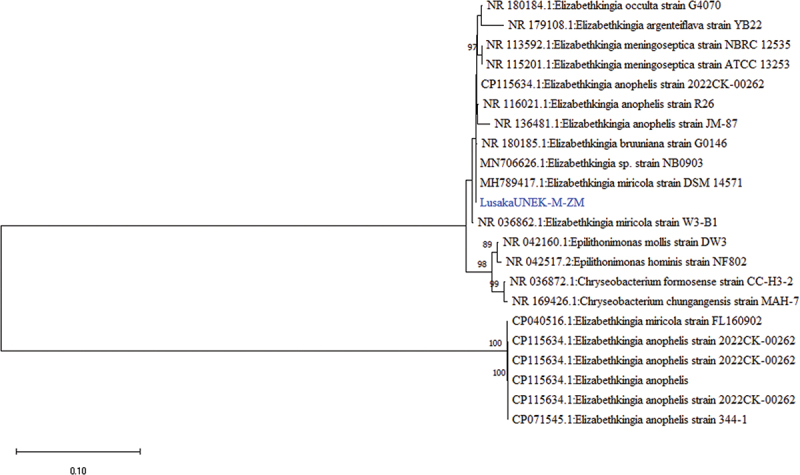
Phylogenetic tree showing evolutionary relationships of the *16S rRNA* gene segment of *Elizabethkingia miricola* (~946 nucleotides in length) from Zambia and reference sequences. The sequence characterised in this study are shown in blue text. Numbers at branch nodes represent bootstrap values ≥60% (based on 1000 replicates). Scale bar represents number of nucleotide substitutions per site.

The *16S rRNA* gene sequence of LusakaUN3EK-M-ZM showed 94.63% identity to several strains of *Elizabethkingia miricola*, recently identified as opportunistic pathogens in humans [42], top of which was *Elizabethkingia miricola* strain DSM_14571 (GenBank: MH7894174). Phylogenetically, LusakaUN3EK-M-ZM was closely related to *Elizabethkingia miricola* (GenBank: MH789417.1) identified in condensed water on the Russian space station [43] (Figure 6).

**Figure 7.**
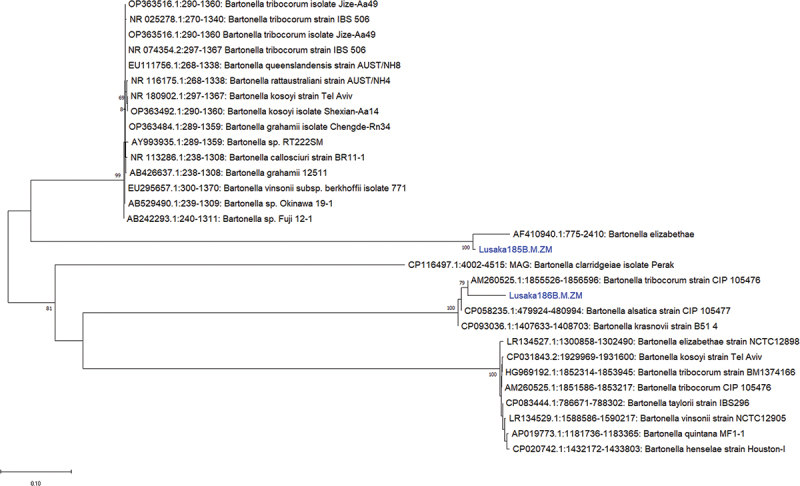
Phylogenetic tree showing evolutionary relationships of the *16S rRNA* gene sequences of *Bartonella* sp (~1676 nucleotides in length) from Zambia and reference sequences. The bartonellae sequence characterised in this study is shown in blue text. Numbers at branch nodes represent bootstrap values (based on 1000 replicates). Scale bar represents number of nucleotide substitutions per site.

In the case of the 16S rRNA nucleotide sequences for Lusaka186BE-M-ZM, it showed 95.72% similarity to *Bartonella triboculum* strain IBS506 from rodents (GenBank: NR_074354.2). The tree topology showed that Lusaka186BE-M-ZM was closely related to *Bartonella triboculum* of an unknown source (Genbank: CIP105476) (Figure 6). Meanwhile, both Lusaka168BE-M-ZM and Lusaka185BE-M-ZM sequences had 100% and 95.72% nucleotide sequence similarity to *Bartonella elizabethae* (GenBank: LR134527), a zoonotic pathogen associated with endocarditis and febrile illness in humans since 1993 [44,45]. The Lusaka185BE-M-ZM 23S rRNA gene sequence formed a distinguishable cluster with *Bartonella elizabethae* (Genbank: AF410940.1) of an unknown source [46] (Figure 7).

**Figure 8.**
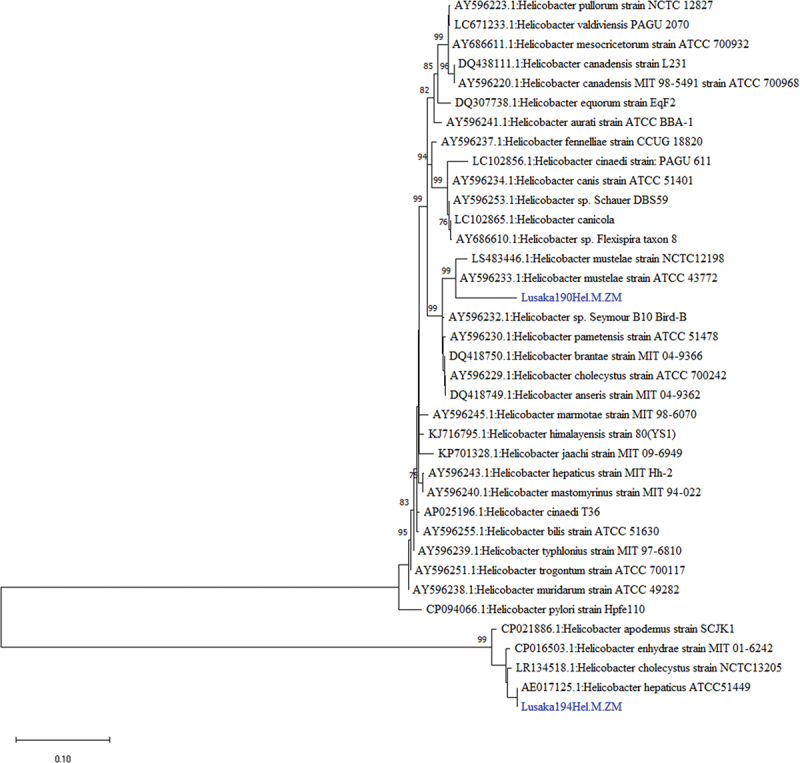
Phylogenetic tree showing evolutionary relationships of the *23S rRNA* gene segment of *Helicobacter* sp (~1747 and 1230 nucleotides in length) from Zambia and reference sequences. Sequences characterised in this study are shown in blue text. Numbers at branch nodes represent bootstrap values ≥60% (based on 1000 replicates). Scale bar represents number of nucleotide substitutions per site.

Two 23S rRNA nucleotide sequences designated Lusaka194Hel-M-ZM and Lusaka190Hel-M-ZM had 94.08% and 97.07% identity to *Helicobacter hepaticus* strain ATCC51449 (GenBank: AY596243.1) and *Helicobacter mustelae* strain NCTC12198 (Genbank: LS483446.1), respectively. Both reference sequences were of unknown origin and host. Phylogenetically, Lusaka194Hel-M-ZM and Lusaka190Hel-M-ZM sequences belonged to distinct clades and were closely related to *Helicobacter hepaticus* (Genbank: AE017125.1) from mice [47] and *Helicobacter mustelae* (Genbank: AY596233.1) from ferrets [48], respectively (Figure 8).

**Figure 9.**
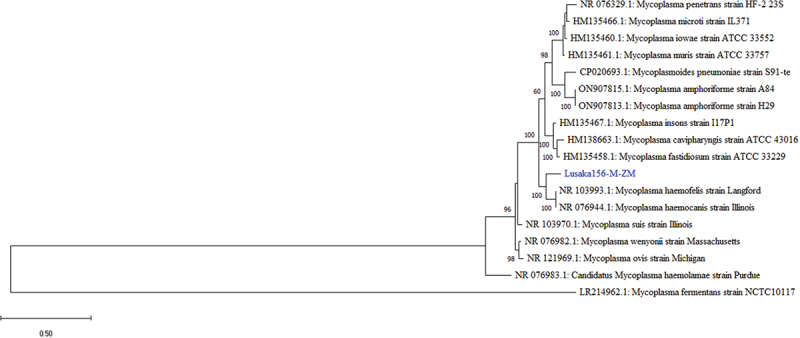
Phylogenetic tree showing evolutionary relationships of the *28S rRNA* gene segment of *Mycoplasma* sp (~1949 nucleotides in length) from Zambia and reference sequences. Sequences characterised in this study are shown in blue text. Numbers at branch nodes represent bootstrap values ≥60% (based on 1000 replicates). Scale bar represents number of nucleotide substitutions per site.

Finally, among the detected bacterial sequences, the *28S rRNA* gene sequence of Lusaka 156-M-ZM showed nucleotide sequence similarity of 81.79% to *Mycoplasma haemofelis* strain Langford-1 (GenBank: FR773153.1) by BLAST search. Phylogenetic analysis of the gene sequence clustered closely to those of *Mycoplasma haemofelis*/Langford-1 (Genbank: NR103993.1) associated with haemolytic anaemia in cats [49] and *Mycoplasma haemocanis*/Illinois (Genbank: NR076944.1) from dogs [50], both from the USA (Figure 9).

#### Parasites

**Figure 10.**
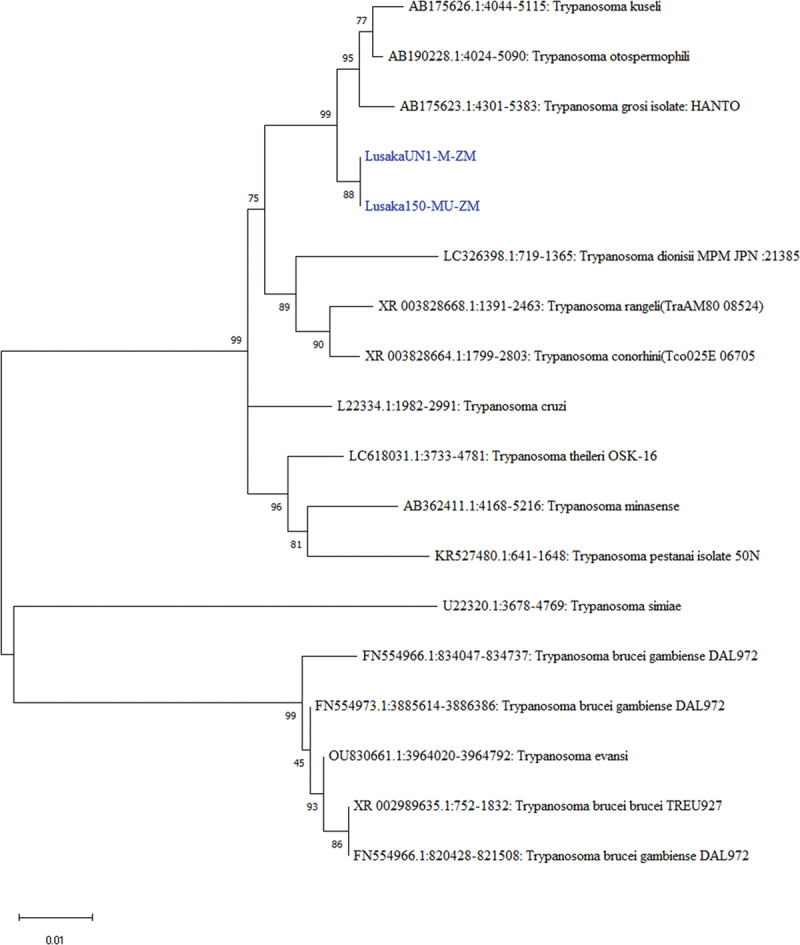
Phylogenetic tree showing evolutionary relationships of the *23S rRNA* gene segments of *Trypanosoma* sp (~1063 and 969 nucleotides in length) from Zambia and reference sequences. The *23S rRNA* gene segments for trypanosomes characterised in this study are shown in blue text. Numbers at branch nodes represent bootstrap values ≥60% (based on 1000 replicates). The scale bar indicates the number of nucleotide substitutions per site.

Besides previously reported nematodes and cestodes [24], two of the *28S rRNA* sequences (LusakaUN1-M-ZM and Lusaka150-MU-ZM) showed 98.32% and 98.36% nucleotide sequence identity to *Trypanosoma kuseli* (GenBank: AB175626.1) and *Trypanosoma otospermophili* (GenBank: AB190228.1) by BLAST analysis, respectively. Phylogenetically, LusakaUN1-M-ZM) and Lusaka 150-MU-ZM sequences clustered together and were closely related to *Trypanosoma kuseli* (Genbank: AB175626.1) and *Trypanosoma otospermophili* (Genbank: AB190228.1) (Figure 10) from squirrels in China and the USA, respectively [51].

**Figure 11.**
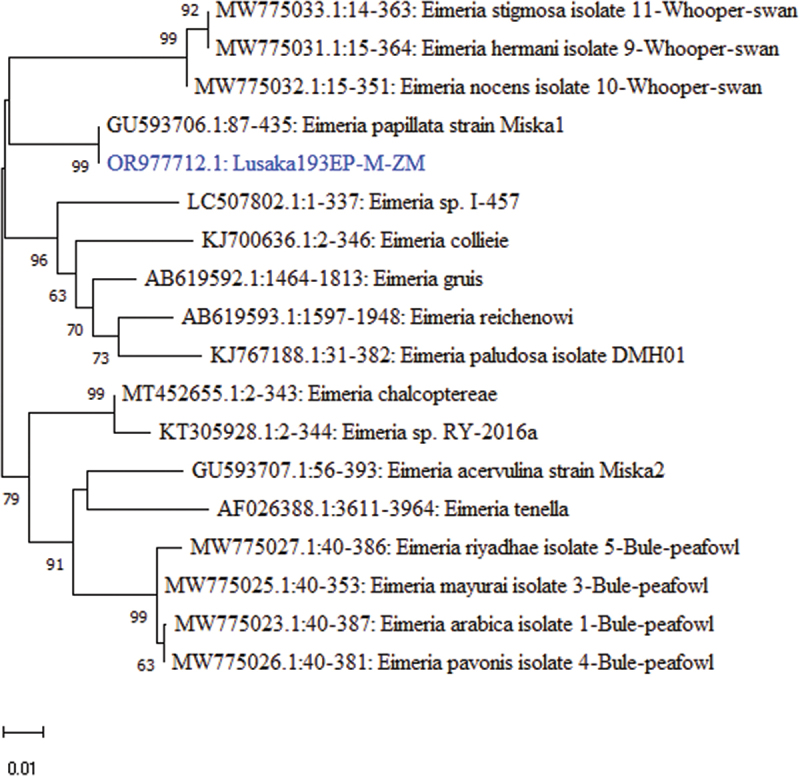
Phylogenetic tree showing evolutionary relationships of the *28S rRNA* gene segments of *Eimeria papillata* (~299 nucleotides in length) from Zambia and reference sequences. Numbers at branch nodes represent bootstrap values ≥60% (based on 1000 replicates). The scale bar indicates the number of nucleotide substitutions per site.

Meanwhile, the *28S rRNA* gene sequence of Lusaka193EP-M-ZM showed 99.71% nucleotide sequence similarity to *Eimeria papillata* strain Miska1 (Genbank: GU593706.1) through BLAST search. Lusaka193EP-M-ZM was phylogenetically related to *Eimeria papillata* strain Miska, a coccidial parasite isolated from the red jungle fowl (Genbank: GU593706.1) (Figure 11).

### Likelihood of occurrence of pathogens/potential pathogens in rodents

A stepwise multivariate model considered the likelihood of a rodent carrying any pathogen in relation to habitat, age, sex, and season. Based on an insignificant Hosmer – Lemeshow goodness-of-fit statistic (*p* = 0.819) and Omnibus Test of Model Coefficients (*p* = 0.000), the model fit the data and was better at predicting variance than the baseline model. The model explained 20.1% (Nagelkerke *R*^*2*^) of the variance in the carriage of all pathogens and correctly classified 72.2% of all pathogen cases. Riverine habitats were identified as a significant predictor (risk factor) of finding pathogens in rodents (OR = 8.45; *p* < 0.001; 95% CI: 3.07–23.26) (Table 2).

**Table 2.** Risk factors and likelihood estimates for the occurrence of any pathogen in rodents.

Risk Factor	Level	OR	95% CI	*p*-value *
Age	Juvenile	9.38	1.47–59.85	0.682
	Adult	Ref		
Sex	Male	1.82	0.90–3.68	0.094
	Female	Ref		
Season	Rainy	1.27	0.61–2.61	0.52
	Dry	Ref		
Habitat	Riverine	8.45	3.07–23.26	<0.001
	Non-riverine	Ref		

Stepwise binary logistic regression; Significance level = *p* < 0.05; OR = Odds ratio; CI = Confidence interval; Ref = Reference category.

## Discussion

Understanding the environmental, host, and pathogen factors that drive infections in reservoir hosts is crucial for unraveling the ecology of pathogens and the mechanisms that could lead to spill-over into humans. This study examined the prevalence of pathogens in rodents, exploring how environmental factors (riverine versus non-riverine areas, season) and host characteristics (age and sex) influenced infection dynamics. By identifying these key factors, the study provided valuable insights into the complex interactions that shaped pathogen transmission and the risks they posed to human health.

### Landscape of pathogens in *M. natalensis*

Bacteria were the most predominant microorganisms as expected while carriage of bacterial pathogens was reported for the first time in rodent semen. Compared to the study conducted in Senegal [18], this study detected a greater number of pathogens, likely due to the use of mNGS. Nevertheless, the findings from both studies underscore the infectious disease risks associated with wild rodents in Africa, extending beyond Lassa fever, leptospirosis, plague, and Q-fever [3,4,8,18,52] and potentially monkey pox [53]. Intriguingly, most pathogens phylogenetically analysed in this study clustered with those from countries far away from Zambia despite a lack of spatio-temporal connections. This suggests possible co-evolution with the hosts or that these pathogens are ubiquitous and have a broad geographical distribution. It may also be because homologous data are not available for geographically and temporally related strains. For example, for *Bartonella* sp., the target gene for most studies has been *gltA* rather than *16S rRNA* used in this study [9].

### Zoonotic significance of reported pathogens

The pathogens identified in this study can be categorised as established disease-causing agents in humans (*Klebsiella michiganensis*, *Klebsiella pneumoniae, Rickettsia* sp., *Bartonella elizabethae,* and *Salmonella enterica*) or emerging disease-causing agents (*Klebsiella variicola, Bartonella tribocorum*, *Elizabethkingia miricola,* and Cardiovirus B). Others can be grouped as pathogens infective to livestock and wild animals (*Helicobacter* sp., *Eimeria papillata*, *Mycoplasma* sp., *Trypanosoma kuseli*, and *Trypanosoma otospermophili*).

*Klebsiella* sp are known for their multi-drug resistance against carbapenem antibiotics [54] and ability to cause severe bloodstream infections in humans particularly among infants [55] and immunocompromised adults [56]. In some cases, bloodstream infections in humans [57,58] may result in mortalities of up to 72% [59]. Meanwhile, some *Klebsiella* sp such as *Klebsiella variicola* have been linked to diarrhoea in humans [60,61] particularly food-borne diarroea as reported in the United Kingdom. Meanwhile, the *K. michiganensis* sequence detected in this study was phylogenetically related to an isolate from a sick child in the USA [55] supporting its potential to jump species. To the best of our knowledge, this is the first report of *K. michiganensis* in wild rodents and particularly in animal foetal tissues anywhere in the world [62]. *K. michiganensis* is a relatively new pathogen discovered in 2012 from a tooth brush holder in the USA [63]. With regards to two *K. pneumoniae* detected in this study, one sequence was phylogenetically related to an isolate from a patient at Johns Hopkins University in the USA based on GenBank records, while another was related to sequences from rabbits in China. The data supports previous reports that *K. pneumoniae* and *K. michiganensis* are widespread and colonise several animals and probably the environment [55]. For that reason, these organims naturally acquire multiple antibiotic resistance genes from varying environments [64].

Similarly, zoonotic *Bartonella* sp present a challenge, as they are linked to clinically complex disease in humans with non-specific symptoms often characterised with fever [45,65,66]. This study detected two bartonellae sequences one of which was related to *Bartonella elizabethae*, a zoonotic infectious disease agent known to be hosted by humans, rodents, and dogs [44,65]. The other sequence was related to *Bartonella tribocorum* which was previously known to infect rodents only until recently when it was linked to disease in humans [66]. These findings are a first in Zambian rodents but a second report of zoonotic bartonella in Zambian wildlife after the discovery of *Bartonella rousetti* in bats [67].

Unlike *Bartonella* sp which are sparsely reported in Zambia, the presence of *Salmonella enterica* in some rodents was not a surprise as this pathogen has been reported in rodents and humans previously [68,69]. Additionally, outbreaks of salmonellosis in humans have also been linked to rodents before in other countries [14]. Therefore, the idea of cross-species transmission of rodent-borne bacterial pathogens is not a fallacy. Moreover, all the *Salmonella enterica* strains detected in this study were related to entero-pathogenic human strains [14]. In the case of *Elizabethkingia miricola*, it was detected for the first time in rodents in Zambia and probably in Africa. Its zoonotic potential is not well defined but has been described as an emerging causative agent of illness in immunocompromised people [42].

The identification of diverse zoonotic pathogens is often concerning especially when novel agents are reported. This was the case of the only *Rickettsia* sp detected herein, Lusaka190RCT-M-ZM, which appears to be a member of the spotted fever group rickettsiae whose geographical spread has increased since the 1980s [70]. The *Rickettsia* sp was related to both *Rickettsia japonica* strain LA16/2015, a spotted fever group rickettsia detected in a human in China (GenBank CP047359.1) and *Rickettsia australis* which causes queensland tick typhus in humans. Spotted fever may manifest as a febrile illness accompanied by headache, malaise, rash, and myalgia [71].

Meanwhile, Chibombo189CV-M-ZM, the only virused detected in this study. formed a distinct lineage basal to Saffold viruses and other Cardiovirus B strains. The phylogenetic tree topology and nucleotide sequence relative to other cardioviruses suggests that Chibombo189CV-M-ZM may be a novel Cardiovirus whose zoonotic potential may be similar to Saffold viruses [72]. Viruses in the genus *Cardiovirus* are single-stranded positive-sense RNA viruses belonging to the family *Picornaviridae*. These viruses have mainly been associated with disease in rodents, but recent evidence has implicated them in respiratory, gastrointestinal, and influenza-like illness in humans [40,72–74]. Considering that this is the second detection of a virus in the genus *Cardiovirus in M. natalensis* in Zambia after the detection of Encephalomyocarditis virus, a type of Cardiovirus A, there is a need for further investigations to understand their ecology and epidemiology in rodents and other wildlife in Zambia [20]. Investigations can be extended to humans presenting with influenza-like symptoms at health facilities, specifically, in areas where the viruses have been reported.

The rest of the other pathogens reported in this study were related to those found in pet animals (*Mycoplasma* sp), mice (*Eimeria papillata* and *Helicobacter* sp), and squirrels (*Trypanosoma kuseli* and *Trypanosoma otospermophili*) [49,51,75–78]. Of veterinary interest was *Mycoplasma* sp. The *Mycoplasma* sp detected in this study appears to be a distinct pathogen with close phylogenetic relationships to both *Mycoplasma haemofelis* from cats and *Mycoplasma haemocanis* from dogs. *Mycoplasma haemofelis* and *Mycoplasma haemocanis* are well known for causing haemolytic anaemia in cats and dogs, but their zoonotic potential is unknown [49,50]. Meanwhile, *Eimeria papillata* is a murine coccidial agent whose contribution to coccidiosis in poultry is not well understood [75,76,79].

### Distribution of pathogens in rodents by organ type

Rodent-borne pathogens are often investigated in liver, kidney, and spleen samples [9–22]. This is the first study to report bacterial pathogens in the semen and foetal tissues of *M. natalensis* or any other rodent species in Africa based on available evidence. The data gives insights into the possibility that semen within seminal vesicles harbours multiple pathogens. The pathogens could have found themselves in semen via infected blood or through faecal contamination during mating. Previously, we reported the presence of rodent-borne viruses in semen housed within seminal vesicles [24]. Therefore, surveillance for pathogens in rodents and other animals could consider semen as an alternative sample alongside liver, kidney, and spleen samples. With regards to infectious potential, it remains to be determined whether bacterial pathogens found in the semen of rodents could be infectious similar to rodent-borne mammarenaviruses [80].

### Possible routes of transmission of pathogens in rodents

The transmission routes and dynamics of maintenance of pathogens among rodents appear to be complex probably relying on host density and presence of chronically infected individuals [19,81], contact with infected urine and faeces [8], and probably habitat type [28]. Among mammarenaviruses, congenital transmission has been suggested [19,24]. *Klebsiella michiganensis*, commonly associated with human preterm infants [54], appears to have the same pathological and transmission mechanisms in rodents as it was only detected in foetal tissues in this study. Such information suggests that *Klebsiella michiganensis* crosses the placenta to infect foetuses congenitally. Therefore, the role of foetuses in the epidemiology of rodent-borne pathogens requires further investigations.

### Riverine areas as potential hotspots for rodent-borne pathogens

Rodents in riverine habitats carried the most pathogens. This could be explained by the possibility of prolonged survival of pathogens due to wet conditions and/or extensive ground cover [82]. The phenomenon can also be explained by the availability of conditions that may favour the survival of rodents and associated population growth resulting in close contact and easy transmission of pathogens. The riverine habitat in Lusaka, from which many pathogens were detected in rodents, was partially disturbed by on and off farming activities. The area had a dense cover of tall Napier grass (*Cenchrus purpureus*) and Rhodesian star grass (*Cynodon nlemfuensis*). On the very edges of the stream and within the water bed, common reeds (*Phragmites* sp) and giant reeds (*Arundo donax*) dominated. The grass was unusually green throughout the year even in the intense summer heat. Giant acacia trees and shrubs dominated the habitat. Sporadic farming activities most likely provided the necessary food for rodents to maintain healthy populations. A hypothetical abundance of permissive hosts in the area probably drove the effective transmission of pathogens. For example, *M.natalensis* rodents were the most abundant small mammals based on trapping success in this study. They fit the description of the most permissive rodent reservoirs [2]. Additionally, since most rodents carrying helminths were found in riverine areas as reported previously [24], the combined impact of wet soil conditions, dense ground cover, and helminths on the prevalence of pathogens in *M. natalensis* cannot be ruled out [83].

### mNGS as a potential tool for surveillance of pathogens

In this study and elsewhere, mNGS proved to be a powerful surveillance tool for a comprehensive assessment of the landscape of pathogens in any sample type [32–35,84–90]. The pathogen-specific nucleotide sequences provided by mNGS were of good quality and long enough for analysis. Impressively, mNGS also successfully detected bacterial and parasitic organisms in this study by targeting the *rRNA* genes. However, differentiation of species within a genus was sometimes challenging as 16S, 23S, and 28S rRNA genes are highly conserved among eukaryotes [91]. Therefore, simultaneous assessment of both RNA and DNA samples whenever blind surveillance for pathogens is conducted using mNGS is necessary. It must be noted that the use of mNGS to investigate the epidemiology of zoonotic pathogens is a recent advancement, as demonstrated in this study and in field research from resource-limited Southeast Asia [86] as well as hospital settings [87,89,90]. However, this method is highly sensitive, precise, and accurate for pathogen detection [92]. Thus, given the high burden of zoonotic diseases and diagnostic challenges in resource-limited countries like Zambia, investing in mNGS infrastructure is crucial for monitoring pathogens every few years to guide prevention and treatment [86].

### Future perspectives

This study underscores the importance of rodents in the epidemiology and surveillance of zoonoses, not only due to the identified pathogens but also due to frequent human-rodent contact and ability to harbour and preserve pathogens in semen and reproductive organs [8].]. Additionally, delineation of hotspots may be vital for cost-effective targeted surveillance of zoonoses using mNGS [85–87].

## Conclusions

This study highlights the significant role that rodents may play in harbouring a diverse array of infectious disease agents with zoonotic potential, many of which are linked to emerging public health threats. The findings underscore the importance of riverine areas as critical hotspots for rodent-borne pathogens in Zambia, suggesting that these regions may present elevated risks for the transmission of diseases to humans, wildlife, and livestock.
